# Relevant In Vitro Predictors of Human Acellular Dermal Matrix-Associated Inflammation and Capsule Formation in a Nonhuman Primate Subcutaneous Tissue Expander Model

**Published:** 2017-01-05

**Authors:** Maryellen Sandor, Patrick Leamy, Pearl Assan, Amardeep Hoonjan, Li-Ting Huang, Marianne Edwards, Wenqi Zuo, Hui Li, Hui Xu

**Affiliations:** LifeCell Corporation, an ACELITY Company, Bridgewater, NJ

**Keywords:** HADM, AlloDerm, DermACELL, Flex HD Pliable, AlloMax

## Abstract

**Objective:** Benchtop methods were evaluated for preclinical inflammation/capsule formation correlation following implantation of human acellular dermal matrices. **Methods:** Dermal matrices were compared with native dermis for structure (histology, scanning electron microscopy), collagen solubility (hydroxyproline), enzymatic susceptibility (collagenase), and thermal stability (differential scanning calorimetry). Results were compared with implantation outcomes in a primate tissue expander model. **Results:** Native dermis, electron beam–sterilized, and freeze-dried human acellular dermal matrices had equivalent morphology, acid-soluble collagen (60.5% ± 6.3%, 65.3% ± 3.2%, and 63.3% ± 2.4%, respectively), and collagenase resistance. Implant results showed minimal inflammation/matrix degradation, lack of capsule formation, insignificant elastic modulus change (57.65 ± 20.24 MPa out-of-package/44.84 ± 23.87 MPa in vivo), and low antibody induction (2- to 8-fold increase) for electron beam–sterilized matrix. Similar results for freeze-dried dermal matrix were previously observed. γ-Irradiated, γ-irradiated/freeze-dried, and ethanol-stored dermal matrices were statistically different from native dermis for acid-soluble collagen (82.4% ± 5.8%, 72.2% ± 6.2%, and 76.8% ± 5.0%, respectively) and collagenase digestion rate, indicating matrix damage. γ-Irradiated matrix-implanted animals demonstrated elevated inflammatory response, foreign body giant cells, capsule formation at the tissue expander junction, and robust matrix metalloproteinase-1 staining with significant elastic modulus decrease (37.43 ± 7.52 MPa out-of-package/19.58 ± 1.16 MPa in vivo). Antibody increase (32- to 128-fold) was observed 6 to 10 weeks following γ-irradiated matrix implantation. Ethanol-stored dermal matrix elicited an acute antibody response (4- to 128-fold increase, 2-4 weeks) and macrophage-concentrated synovial-like hyperplasia at the tissue expander junction, moderate matrix metalloproteinase-1 staining, and significant elastic modulus decrease (61.15 ± 9.12 MPa out-of-package/17.92 ± 4.02 MPa in vivo) by 10 weeks implantation. **Conclusion:** Demonstrated loss of collagen integrity in vitro may be predictive of inflammation/capsule formation in primate tissue expander models. These results may be further predictive of clinical observations.

In recent years, women undergoing breast reconstruction postmastectomy are experiencing a greater rate of successful outcomes due to advancement in technique and increased surgeon expertise, as well as the wide selection and improvement in materials for use in these procedures. Breast reconstruction, which requires part of the breast to be recreated from autologous flaps (transverse rectus abdominis, deep inferior epigastric perforator, latissimus dorsi)^1^^,^^2^ or synthetic materials due to loss of native tissue, requires careful circumvention of multiple potential complications to achieve a successful outcome. General complications include seroma or hematoma formation, wound infection, implant extrusion, skin flap necrosis, and capsular contracture,^3^^,^^4^ with the rate of capsular contracture for implant-based reconstruction being reported as 10% to 15%.^5^^-^7 The incidence of complications and severe contracture may lead to the need for implant removal and revision surgery.^8^^-^10

The cause of capsular contracture has been hypothesized to be due to numerous different factors,^11^ including subclinical infections leading to capsule formation and fibrosis,^12^ chronic inflammatory cellular environment around the implant^5^^,^^13^ based on observations of hypertrophic scarring,^14^ or unsuitable surface topography of the synthetic implant.^15^^,^^16^ Each of these, as well as capsule formation itself, a necessary precursor to capsular contracture,^17^^,^^18^ has been clearly linked to the presence^5^^,^^12^^,^^13^ and degree of inflammation.^19^

Implantation of acellular dermal matrices (ADMs) for support of the lower breast pole has been increasingly used in reconstructive procedures in recent years, and examination of associated outcomes generally supports its usage.^20^^-^22 Human acellular dermal matrix (HADM) implantation, however, can either contribute incrementally to the implant-based inflammatory response or aid in inhibiting such a response as demonstrated in cell culture and preclinical studies.^23^^-^25 There are currently dozens of commercially available ADMs, and previous studies have suggested that use of particular HADMs in conjunction with a tissue expander (TE) may be useful for reducing capsule formation and fibrosis around breast implants^24^^,^^25^ whereas other HADMs may not be as effective.^25^ It is hypothesized that minimizing inflammatory response to HADMs and, subsequently, to the adjacent synthetic implants, is dependent on avoiding the implantation of material containing inflammation-inducing immunoantigens or damaged matrix proteins, the former of which have been shown to elicit local and systemic immune responses^26^ and the latter of which has resulted in foreign body response consisting of inflammation, capsule formation, and HADM resorption.^25^

It is therefore critical for the surgeon to make an informed decision in selecting an appropriate ADM for use in his or her patients. To this end, thorough testing has been performed in an attempt to differentiate the numerous materials, which have been manufactured using unique processing, sterilization, and storage methods and reveal different perspectives on the content and function of the various available ADMs. Sorting through the relevant data, however, can be complex since it is critical for the test methods employed to distinguish proinflammatory from anti-inflammatory HADMs. While in vitro evaluations of these materials have included testing for specific growth factors, DNA content,^27^^,^^28^ collagen stability,^29^ antibiotic content,^30^ or mechanical properties, 31^,^^32^ it is of critical importance to note which of these assessments correlate to downstream functional outcomes beyond the perioperative period, especially as it relates to the potential for inflammatory response.^23^ Correlation to successful outcomes in an animal model, particularly in conjunction with a synthetic TE,^13^^,^^33^ and even more importantly in an immunologically similar model to humans such as a primate,^24^^,^^25^ is particularly useful. In this study, we aim to correlate in vitro morphological, biochemical, and biophysical analyses of several commercially available HADMs with the resulting host response related to inflammation, capsule formation, and HADM remodeling in a nonhuman primate subcutaneous TE model and to determine properties of tissues that may be predictive of preclinical and, potentially, clinical outcomes. Benchtop tests were chosen on the basis of their ability to demonstrate collagen damage/nativity, which has been shown to have a significant impact on inflammatory host response in vivo.^29^^,^^34^

## METHODS

### Materials

Five commercially available HADMs were evaluated through a series of in vitro tests: an aseptically processed freeze-dried HADM (HADM-FD) (AlloDerm; LifeCell, an ACELITY Company, Bridgewater, NJ) and an equivalent product sterilized using electron beam (e-beam) irradiation and provided hydrated (e-HADM, AlloDerm Ready-to-Use; LifeCell, an ACELITY Company, Bridgewater, NJ), a γ-irradiated HADM (g-HADM) (DermACELL; LifeNet, Virginia Beach, Va), an aseptically processed HADM stored in ethanol (EtOH-HADM) (Flex HD Pliable; MTF, Edison, NJ), and a γ-irradiated freeze-dried HADM (g-HADM-FD) (AlloMax; CR Bard, Murray Hill, NJ). Each of these products was compared with native unprocessed human cellular dermal matrix (HCDM), which served as a control material.

### Out-of-Package HADM in Vitro Analysis

#### HADM in vitro analysis sampling

Unless otherwise noted, all in vitro tests were performed on 3 replicate samples from each of 4 distinct lots for each HADM material and native HCDM. Multiple lots were used in an effort to reduce any potential effect of donor-to-donor variability.

#### Morphological and structural evaluation

Morphological characterization was performed as previously described.^29^ Briefly, a single 1 × 1-cm out-of-package sample of each HADM was rinsed in 0.9% saline for 1 hour, followed by fixation in either 10% neutral buffered formalin for histological evaluation or 2% glutaraldehyde for scanning electron microscopy (SEM). Following fixation, each sample was subjected to dehydration in a series of graded ethanol solutions, ranging from 50% up to 100%. For histological evaluation, samples were then embedded in paraffin and sectioned to a thickness of 5 μm using a diamond knife and stained using a standard hematoxylin and eosin (H&E) method, before being cover-slipped and evaluated for collagen morphology under bright-field microscopy by a blinded histopathologist. For SEM, dehydrated samples were transferred to 100% hexamethyldisilazane for drying, followed by mounting and sputter-coating with gold-palladium under vacuum conditions (<8 Pa) for 120 seconds. Cross-sectional micrographs of HADMs were captured with a desktop scanning electron microscope (NeoScope JCM-5000; Jeol Ltd, Tokyo, Japan) at 10 kV.

#### Hydroxyproline assay for collagen content

A modified hydroxyproline assay was used to determine acid-soluble collagen content of the HADM samples as previously described.^35^ All chemicals were purchased from Sigma-Aldrich (St Louis, Mo) or Thermo-Fisher (Waltham, Mass) unless otherwise noted. Briefly, HADM samples (approximately 20-mg dry weight) were incubated in 1 mL of chloroform for 16 to 20 hours, followed by decantation and freeze-drying. Acid hydrolysis was performed by adding 1 mL 6N HCl and heating at 95°C for 4 to 6 hours, until fully digested. Digested samples were then diluted 1:20 with distilled water, and a 50-μL aliquot of the diluted sample transferred to a 24-well plate with the additions of 100 μL 2-propanol and 50 μL 6 mM Chloramine-T in acetate-citrate buffer. Plates were incubated at room temperature for 20 minutes, followed by the addition of 1.2 mL of modified Ehrlich's reagent (2 g *p*-dimethylaminobenzaldehyde in 3 mL 70% perchloric acid and 28 mL 2-propanol solution) and additional incubation for 25 minutes at 60°C for color development. Trans-4-hydroxy-l-proline (hydroxyproline) solution standards, ranging from 0 to 180 μg/mL, were treated identically to samples and used to create a standard curve. Reaction products for standards and samples were measured at 560 nm by a spectrophotometer. Hydroxyproline content was determined from the regression line of the standard curve, and total acid-soluble collagen content calculated on the basis of a presumed collagen composition of 12.5% hydroxyproline.

#### Collagenase susceptibility assay

Susceptibility to in vitro digestion by collagenase was determined using an assay as previously described.^29^ Briefly, HADM samples (∼100 mg) were rinsed in 0.9% saline, followed by the addition of 1.5 mL 10 mM Tris-HCl buffer with 2 mM CaCl_2_ (pH 7.5) and 100 U/mL of collagenase (Sigma-Aldrich, St Louis, Mo). Following incubation at 37°C for 0, 4, 8, 12, and 16 hours, samples were centrifuged, washed with distilled water, and freeze-dried. Percentage dry mass remaining was used to determine the susceptibility of the samples to enzymatic digestion and as an indirect measure of collagen nativity.

#### Thermal analysis

For differential scanning calorimetry (DSC), HADM samples (10-25 mg) were rinsed in phosphate buffered saline (PBS) and hermetically sealed in aluminum crucibles as previously described.^29^ Samples were heated at a rate of 3°C/min, from 2°C to 120°C, using a differential scanning calorimeter (model Q2000; TA Instruments, New Castle, Del), and thermograms analyzed with Universal Analysis 2000 software to determine the collagen denaturation temperature onset (*T*_o_) and enthalpy (Δ*H*) of melting.

### Nonhuman Primate Model

#### Study design

All in vivo work was conducted per Institutional Animal Care and Use Committee (IACUC) approved protocol. Twelve adult male Caribbean vervets (*Cercopithecus aethiops*), weighing between 4 and 6 kg, were included in the study. Animals were randomly assigned to 1 of 3 treatment groups (4 animals per group), each to receive along the midline of the dorsum a subcutaneous TE implant. Animals were randomized to receive either (1) TE with an overlay of e-beam–irradiated HADM (e-HADM + TE); (2) TE with an overlay of γ-irradiated HADM (g-HADM + TE); or (3) TE with an overlay of ethanol-soaked HADM (EtOH-HADM + TE). At 10 weeks following implantation, animals were euthanized and soft tissue around the TE was harvested. TE with an overlay of freeze-dried HADM (HADM-FD + TE) and TE with an overlay of γ-irradiated, freeze-dried HADM (g-HADM-FD) were previously implanted in this same animal model in a previous study^25^ and so were not implanted duplicatively here.

#### HADM-TE implantation procedure

The TE implantation procedure was performed as previously described in the literature.^24^ Briefly, animals were fasted for 24 hours prior to the procedure and anesthetized by intramuscular (IM) injection of ketamine (10 mg/kg) and xylazine (1.0 mg/kg) and prophylactic antibiotic cefazolin sodium (125 mg). Once anesthetized, each animal was placed in the prone position and its upper back was shaved and aseptically prepared for surgery. One horizontal curved incision, approximately 3 cm, was made through the skin and subcutaneous tissues on the back below the shoulder blades. A subcutaneous pocket was created just above the dorsal musculature and deep fascia on either side of the spine to accommodate a 30-mL circular, smooth silicone shell TE with remote microport, 3 cm in diameter (PMT Corp, Chanhassen, Minn). For each animal, a single 6 × 6-cm dermal graft sheet was placed into the subcutaneous pocket and sutured down in a circumferential pattern to the underlying dorsal musculature using 2-0 polypropylene suture in a continuous pattern. A TE was inserted between the muscle and the dermal graft and positioned such that it was completely covered by the dermal graft with the exception of the deep surface where the implant was placed directly against the underlying muscle and then filled with 25-mL sterile saline solution. The port and the tubing were then tied off and removed and the TE anchored to the fascia with 2-0 polypropylene sutures around the port remnant. The subcutaneous layer was then closed with polydioxanone suture in a continuous subcuticular pattern. The skin was closed using nonabsorbable nylon sutures in an interrupted pattern. The animals were given a 3-day course of banamine (2.0 mg/kg, IM) (Schering-Plough Animal Health, Kenilworth, NJ) or buprenorphine (0.01 mg/kg, IM) immediately following surgery. This analgesia regimen was continued for up to 3 days postoperatively if signs of pain were observed. All animals underwent biweekly physical examinations to monitor for complications, particularly at the surgical site.

#### Tissue harvesting

All animals were euthanized by intravenous injection of pentobarbital. TE and surrounding tissues were removed en bloc, inclusive of the HADM material, subcutaneous tissues above, and the thoracic muscle below the implanted TE as previously described.^24^^,^^25^ The entire explanted surgical pocket was then placed in ice-cold RPMI (Roswell Park Memorial Institute) medium solution and shipped overnight on ice for subsequent analysis. Once received, a central 1-cm wide tissue strip, spanning the entire width of the implanted HADM, was collected for immediate mechanical analysis while the remaining lateral segments were fixed in 10% formalin for histological and immunohistochemical analyses.

### Postexplant Testing

#### Histological and immunohistochemical staining

For postexplant testing, samples taken from the apex of the TE were prepared for H&E histology and immunohistochemical staining as previously described.^25^ Briefly, paraffin-embedded HADMs were deparaffinized, rehydrated, and proteinase K applied for antigen retrieval. Mouse anti-human monoclonal antibodies to smooth muscle cell alpha-actin (α-SMA; Sigma cat #A5691) and CD-68 (Zymed/Invitrogen cat#08-0125), as well as rabbit antibody to matrix metalloproteinase-1 (MMP-1; Fitzgerald cat#10R-M112a), were applied using the appropriate dilutions. Detection was achieved using secondary anti-murine (Biorad cat#170-6516) or anti-rabbit (Thermo Shandon cat# TL-060-HL) immunoglobulin G (IgG) conjugated with horseradish peroxidase, and labeling was visualized with diaminobenzidine. Routine H&E staining was evaluated for the overall presence of capsule, infiltrating fibroblasts, and inflammatory response. Immunohistochemical staining was evaluated for the presence, location, and intensity of specific staining. Anti-CD-68, anti-α-SMA, and anti-MMP-1 staining was specifically evaluated to assess the degree of inflammatory/foreign body response (macrophages), capsule/scar formation (myofibroblasts), and collagenase enzymatic activity, respectively.

#### Antibody titer in response to HADM implantation

To determine the systemic immune response to HADMs, samples were rinsed with 0.9% saline, freeze-dried, and micronized using a cryo-mill with liquid nitrogen as previously described.^26^ Micronized tissue powder was suspended in PBS, applied as a coating to Immulon 96-well polystyrene microtiter plates (Thermo Fisher Scientific, Waltham, Mass), and air-dried overnight. Serum samples were taken from each animal at the following time points: just prior to implant (time 0) and at 1, 2, 4, 6, 8, and 10 weeks following implantation. Serum serial dilutions were incubated in coated microtiter wells for 2 hours. After washing, the plates were then incubated for 1 hour with goat anti-human IgG conjugated to alkaline phosphatase. For antibody detection, plates were rinsed and incubated with *p*-nitrophenyl phosphate substrate for 1 hour. Reaction product was measured at 405 nm by a spectrophotometer, and increase in bound antibody titer by reaction to implanted HADM was determined by comparison with the time 0 time point using the serial dilution curves.

#### Biomechanical testing

Out-of-package HADM samples (1 × 5-cm strips) were prepared per manufacturer's instructions and subjected to uniaxial tensile testing on an Instron material tester (model 5865; Instron, Norwood, Mass), equipped with pneumatic grip designed for soft tissues and 1-kN load cell as previously described.^25^ Sample thickness was measured using a laser micrometer before testing. Testing parameters included a 4-cm gauge length and a controlled strain rate of 1.65 mm/min. Central 1-cm wide tissue strips, spanning the entire width of the implanted HADM, were collected from the explanted TE sites and tensile tested in an identical manner to determine the effect of implantation on mechanical properties. Any observable host tissue adhered to the HADM was removed by blunt dissection before testing. Mechanical data were collected using Bluehill software. Mechanical parameters of maximum stress (MPa) and elastic modulus (MPa) were compared using Student's *t* test. Comparisons were made between out-of-package commercial product and native HCDM as well as between out-of-package and postimplant mechanics for each commercial product.

#### Statistical analysis

Unless otherwise noted, in vitro tests were run on triplicate samples from each of 4 distinct HADM lots. Mean and standard deviation from each group of 4 tested lots are reported. Student's *t* test was used to determine significance between each HADM product and native dermis (HCDM) for collagen solubility/hydroxyproline assay, collagenase susceptibility assay, DSC thermal analysis results, and biomechanical testing, both out-of-package and postimplant. Additional comparisons were also made between HADM products, when appropriate.

## RESULTS

### Out-of-package HADM in vitro analysis

#### Morphological and structural evaluation

**Figure 1:**
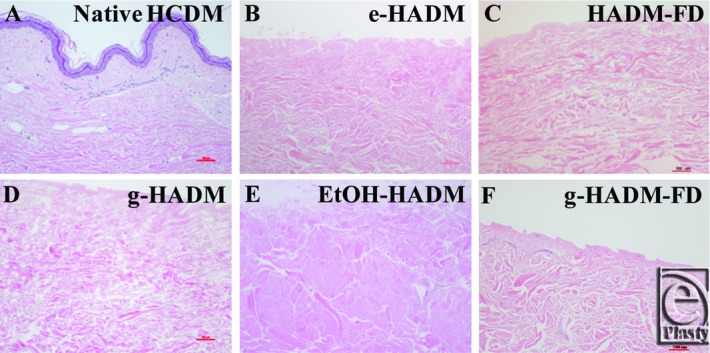
Histological out-of-package morphology of HADMs. Representative out-of-package hematoxylin and eosin histological staining of HADMs as compared with native human dermis (100×). (a) Native HCDM with cellular dermis and attached epidermis, (b) electron beam–irradiated HADM (e-HADM), (c) freeze-dried HADM (HADM-FD), (d) γ-irradiated HADM (g-HADM), (e) ethanol-stored HADM (EtOH-HADM), and (f) γ-irradiated/freeze-dried HADM (g-HADM-FD). HADM indicates human acellular dermal matrix; HCDM, human cellular dermal matrix.

Basic histological evaluation of HADM structure in comparison with native HCDM revealed few differences (Fig 1). The majority of HADMs maintained the native fibrillar morphology of the reticular layer and the distinct structure of the papillary layer, similar to unprocessed human skin, save for EtOH-HADM (Fig 1*e*), which exhibited an amorphous collagen structure, likely due to its origin from a deeper layer of the dermis than the other commercial products. None of the HADMs showed evidence of intact cellular remnants postprocessing.

**Figure 2:**
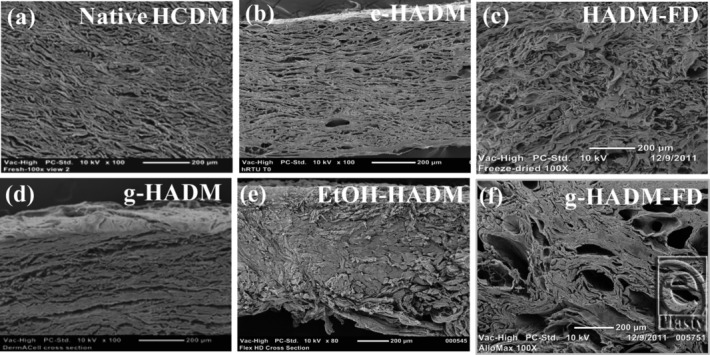
Ultrastructural out-of-package morphology of HADMs. Representative out-of-package scanning electron micrographs of HADMs as compared with native human dermis (100×). (a) Native HCDM, (b) electron beam–irradiated HADM (e-HADM), (c) freeze-dried HADM (HADM-FD), (d) γ-irradiated HADM (g-HADM), (e) ethanol-stored HADM (EtOH-HADM), and (f) γ-irradiated/freeze-dried HADM (g-HADM-FD). HADM indicates human acellular dermal matrix; HCDM, human cellular dermal matrix.

Ultrastructural analysis of HADMs revealed similar structure among native HCDM, e-HADM, and HADM-FD (Figs 2*a*–2*c*), which demonstrated a fibrillar mesh-like morphology, with pores reminiscent of preserved vasculature scattered throughout these matrices. In contrast, the 3 other commercial HADMs demonstrated various structural differences from native HCDM, with g-HADM having a somewhat fibrillar, slightly compacted structure (Fig 2*d*); EtOH-HADM having a condensed, nonporous structure (Fig 2*e*); and g-HADM-FD exhibiting an abundance of large voids, dissimilar in size to native vasculature, and surrounded by compressed collagen fibers (Fig 2*f*).

#### Hydroxyproline assay for collagen content

**Figure 3:**
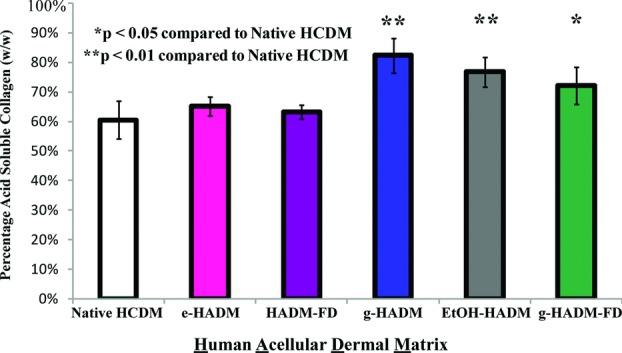
In vitro collagen solubility of HADMs. Relative acid solubility of HADMs as determined by hydroxyproline assay. Percentage of collagen solubilized in 6N HCl was determined by Chloramine-T assay for hydroxyproline content. Data are presented as percentage solubility on a weight/weight basis. Each bar represents 3 replicates taken from each of 4 distinct HADM lots. (*) *P* < .05 compared with native dermis; (^**^) *P* < .01 compared with native dermis. HADM indicates human acellular dermal matrix; HCDM, human cellular dermal matrix; e-HADM, electron beam–irradiated HADM; HADM-FD, freeze-dried HADM; g-HADM, γ-irradiated HADM; EtOH-HADM, ethanol-stored HADM; and g-HADM-FD, γ-irradiated/freeze-dried HADM.

Acid-soluble collagen content of HADMs, determined by hydroxyproline assay, varied from 63.3% ± 2.4% for HADM-FD to 82.4% ± 5.8% for g-HADM (Fig 3). The amounts of soluble collagen for both HADM-FD and e-HADM (65.3% ± 3.2%) were not significantly different from each other or from native HCDM (60.5% ± 6.3%). The other 3 HADMs in this study exhibited acid-soluble collagen fractions significantly different from native HCDM, indicating an increase in denatured or unraveled collagen and correlating with the altered morphologies observed by SEM: g-HADM (82.4% ± 5.8%, *P* < .01), EtOH-HADM (76.8% ± 5.0%, *P* < .01), and g-HADM-FD (72.2% ± 6.2%, *P* < .05).

#### Collagenase susceptibility assay

**Figure 4:**
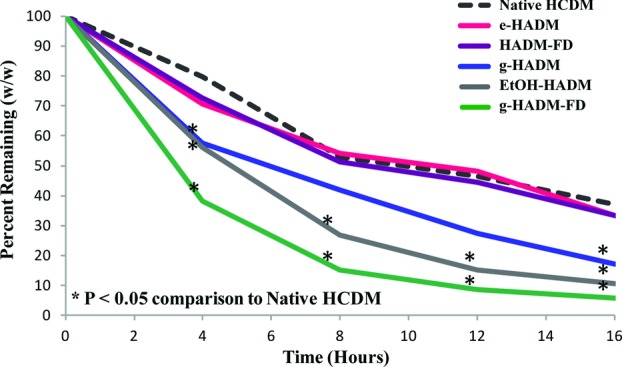
In vitro susceptibility of HADMs to digestion by collagenase. Percentage weight remaining for HADM samples as compared with native HCDM when subjected to partial in vitro collagenase digestion over time. Each curve represents 3 replicates taken from each of 4 distinct HADM lots. (*) represents a statistical difference from native HCDM (Student's *t* test, *P* < .05). There were no statistical differences between e-HADM and native HCDM, between HADM-FD and native HCDM, or between e-HADM and HADM-FD. HADM indicates human acellular dermal matrix; HCDM, human cellular dermal matrix; e-HADM, electron beam–irradiated HADM; HADM-FD, freeze-dried HADM; g-HADM, γ-irradiated HADM; EtOH-HADM, ethanol-stored HADM; and g-HADM-FD, γ-irradiated/freeze-dried HADM.

Resistance to enzymatic digestion by collagenase over time was also used as an indirect measure of collagen nativity. Comparison of the digestion rate of commercial HADMs with that of native HCDM revealed a lack of statistical difference between e-HADM, HADM-FD, and native HCDM at any of the evaluated time periods (Fig 4). Statistical analysis showed a significant difference between g-HADM and native HCDM at 4 and 16 hours, as well as between EtOH-HADM, g-HADM-FD, and native HCDM at each of the evaluated time points, indicating a change in collagen structure, allowing for more rapid enzymatic digestion.

#### Thermal analysis

**Figure 5:**
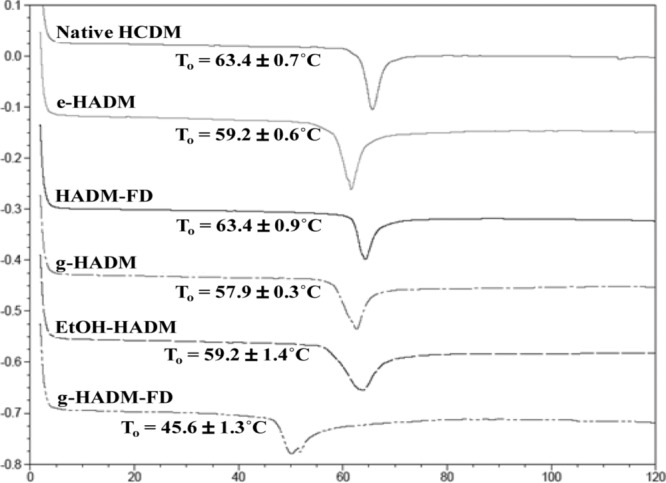
Out-of-package thermal stability of HADMs. Representative differential scanning calorimetric thermograms of HADMs as compared with native HCDM. Onset of melting temperature (*T*_o_) for each HADM is shown on the respective thermograms. Each curve represents 3 replicates taken from each of 4 distinct HADM lots. Width of melting peak was also evaluated and compared with native HCDM as a measurement of matrix change. HADM indicates human acellular dermal matrix; HCDM, human cellular dermal matrix; e-HADM, electron beam–irradiated HADM; HADM-FD, freeze-dried HADM; g-HADM, γ-irradiated HADM; EtOH-HADM, ethanol-stored HADM; and g-HADM-FD, γ-irradiated/freeze-dried HADM.

Thermal analysis by DSC yielded similar collagen onset melting/denaturation temperatures (*T*_o_) for a majority of commercial HADMs in this study, ranging from 57.9°C **±** 0.3°C (g-HADM) to 63.4°C **±** 0.9°C (HADM-FD), with HADM-FD demonstrating properties equivalent to native HCDM (*T*_o_ = 63.4°C **±** 0.7°C) (Fig 5). Of the commercial products, only g-HADM-FD exhibited an onset melting temperature that was substantially lowered from that of native HCDM at 45.6°C **±** 1.3°C, suggesting that the collagen in this HADM has been significantly changed or damaged. In addition, the thermal profiles (Fig 5) indicated differences in collagen stability, with e-HADM and HADM-FD having equivalently narrow melting peaks, similar to native HCDM. Melting peaks for g-HADM and EtOH-HADM, despite having similar *T*_o_, were significantly broader, indicating a change in collagen from native HCDM. The DSC thermogram for g-HADM-FD exhibited the broadest melting peak of the commercial products tested.

### Postexplant testing

All animals implanted with HADM and TE survived to the 10-week explant date without major complications.

#### Histological and immunohistochemical staining

**Figure 6:**
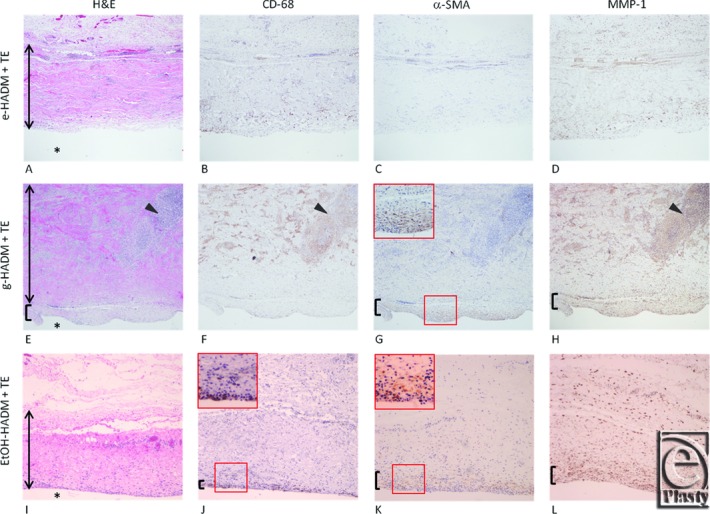
Histological host response to HADMs following 10 weeks’ implantation. Representative serial tissue sections overlying TE interface (*) for e-HADM + TE (a–d); g-HADM + TE (e–h); EtOH-HADM + TE (i–l). hematoxylin and eosin (a, e, i); anti–CD-68/macrophages (b, f, j); α-SMA/myofibroblasts (c, g, k); MMP-1/collagenase (d, h, l). Vertical arrows indicate HADM thickness. Arrowheads indicate macrophage (e, f) colocalization with MMP-1 expression (h) for g-HADM. Brackets indicate α-SMA–positive capsule formation (e, g, inset) and MMP-1 (h) for g-HADM, and synovial-like metaplasia (j, inset), overlaid by α-SMA (k, inset) and MMP-1–positive capsule (l), for EtOH-HADM. e-HADM demonstrated lack of capsule formation (a, c), minimal macrophages (b), and minimal MMP-1/degradation (d). HADM indicates human acellular dermal matrix; e-HADM, electron beam–irradiated HADM; TE, tissue expander; g-HADM, γ-irradiated HADM; EtOH-HADM, ethanol-stored HADM; α-SMA, smooth muscle cell alpha-actin; and MMP-1, matrix metalloproteinase-1.

HADM tissue samples (e-HADM, g-HADM, and EtOH-HADM), explanted from the apex of the TE following 10 weeks’ subcutaneous implantation, were evaluated for cellular infiltration, inflammatory response, capsule presence, and degree and distribution of MMP-1. Descriptions of the histological findings were generated in a blinded manner. Representative histophotomicrographs are shown in Figures 6*a*-*l*.

H&E staining revealed widespread cellular infiltration for e-HADM (Fig 6*a*), with distribution of fibroblasts and numerous blood vessels of various sizes throughout the matrix. There was a lack of capsule-like structure observed at the HADM-TE interface. A low-level inflammatory response was also observed and confirmed via anti–CD-68 immunostaining for macrophages (Fig 6*b*) near the e-HADM–TE junction. Staining for α-SMA positively identified several large blood vessels (Fig 6*c*), whereas the lack of myofibroblast-like cell detection at the TE junction confirmed the lack of capsule formation. The presence of MMP-1, or collagenase, appeared lightly scattered throughout the e-HADM section (Fig 6*d*) and generally colocalized with the CD-68–positive macrophage population (Fig 6*b*).

In contrast, the robust cellular infiltration into g-HADM, observed with H&E staining (Fig 6*e*), was predominantly inflammatory in nature, with hypercellular areas of CD-68–positive macrophages and foreign body giant cells throughout the upper portion of the histological section (Fig 6*f*). Confirmatory staining for α-SMA–positive myofibroblasts (Fig 6*g*) revealed the capsular nature of the rippled tissue layer at the g-HADM–TE junction, which also showed lighter staining with H&E (Fig 6*e*), indicating new collagen deposition at this interface. MMP-1 staining (Fig 6*h*) was widespread, with heavily concentrated areas coincident with both the capsular structure and inflammatory cell populations.

EtOH-HADM demonstrated widespread fibroblast-like cell infiltration into the matrix and several large blood vessels by H&E staining (Fig 6*i*), with moderate inflammatory cell presence and positive staining of macrophages concentrated at the EtOH-HADM junction as in synovial-like metaplasia (Fig 6*j*). α-SMA–positive staining was observed just superficial to the band of macrophages, indicating capsule formation (Fig 6*k*). Moderate levels of MMP-1 staining were observed coincident with the capsule/synovial-like metaplasia and also scattered throughout the EtOH-HADM (Fig 6*l*).

#### Antibody titer in response to HADM implantation

**Figure 7:**
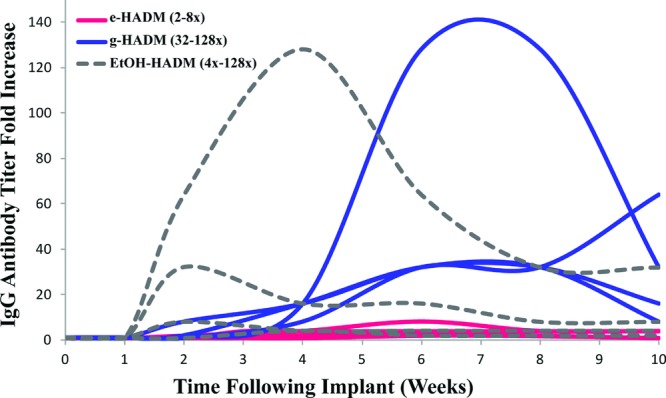
Systemic antibody induction as represented by serum IgG fold increase following subcutaneous HADM implantation in conjunction with a tissue expander. Each curve represents an individual animal, and serum IgG increase is represented as fold increase as compared with baseline (preimplant) levels for each individual animal. Animals receiving e-HADM showed a 2- to 8-fold range increase in antibody titer, animals receiving g-HADM showed a 32- to 128-fold increase, and EtOH-HADM–implanted animals showed a 4- to 128-fold increase. HADM indicates human acellular dermal matrix; IgG, immunoglobulin G; e-HADM, electron beam–irradiated HADM; g-HADM, γ-irradiated HADM; and EtOH-HADM, ethanol-stored HADM.

Nonhuman primate serum was tested for antibodies formed in response to HADM materials (e-HADM, g-HADM, EtOH-HADM) implanted in a subcutaneous TE model for 10 weeks. As compared with a preimplant antibody level within each animal, IgG antibody titers increased a maximum of 2- to 8-fold for e-HADM, 32- to 128-fold for g-HADM, and 4- to 128-fold for EtOH-HADM (Fig 7). Antibody titers peaked at around 3 to 6 weeks for e-HADM, at 6 to 10 weeks for g-HADM, and at 2 to 4 weeks for EtOH-HADM. This suggests a greater induction of IgG antibodies in response to implantation of g-HADM and EtOH-HADM, as compared with e-HADM, and a delayed induction in response to g-HADM in comparison with the other 2 HADMs.

#### Biomechanical testing

Commercial HADMs were tested for mechanical properties both prior to and following 10 weeks subcutaneous implantation with a TE. Native HCDM was also evaluated. Since commercial HADM products range in thickness, maximum stress (strength), and elastic modulus, material properties independent of thickness, were evaluated for comparison across product types and individual donor lots.

Of the 3 HADMs tested in the nonhuman primate model, only EtOH-HADM had significantly higher (*P* < .0005) out-of-package maximum stress (strength) (19.63 ± 3.01 MPa) than native HCDM (12.20 ± 3.51 MPa), whereas e-HADM and g-HADM had equivalent maximum stress (strength) (12.96 ± 4.65 MPa and 11.87 ± 2.02 MPa, respectively) (Table 1). The out-of-package elastic modulus was equivalent among native HCDM (52.17 ± 11.98 MPa), e-HADM (57.65 ± 20.24 MPa), and EtOH-HADM (61.15 ± 9.12 MPa). Interestingly, e-HADM and g-HADM, which were similar in strength out-of-package, were significantly different from one another with respect to elastic modulus, with significantly lower resistance to stretch for g-HADM (37.43 ± 7.52 MPa), indicating additional differences between these 2 HADMs.

Each of the commercial HADMs in this study decreased significantly in maximum stress (strength) following 10 weeks implantation (Table 1). Although EtOH-HADM was shown to be significantly stronger out-of-package than the other materials, it resulted in the weakest postexplant strength (5.21 ± 1.57 MPa), retaining only 26.51% of its original strength following 10 weeks implantation, whereas e-HADM and g-HADM retained (7.62 ± 2.19 MPa) 58.83% and (7.90 ± 1.09 MPa) 66.58% of their original strength, respectively. EtOH-HADM and g-HADM also demonstrated statistically significant decreases in elastic modulus following 10 weeks implantation to (17.92 ± 4.02 MPa) 29.30% and (19.58 ± 1.16 MPa) 52.31%, respectively, whereas e-HADM exhibited a nonsignificant decrease, retaining (44.84 ± 23.87 MPa) 77.78% of its original elastic properties following 10 weeks implantation.

## DISCUSSION

Capsule formation is the body's normal response to the implantation of a foreign object, such as a breast implant or pacemaker.^36^ Capsular contracture, however, being a common cause of breast reconstruction revision surgery,^37^^,^^38^ occurs mostly in context of breast implant complications due to the specific immune/inflammatory response to the implanted foreign materials.^17^^,^^36^ Infiltrating inflammatory cells, unsuccessfully attempting to degrade the permanent implant, will lead to an increase in inflammatory cytokines, tissue remodeling factors such as metalloproteinases (MMPs), and other growth factors,^36^^,^^39^^,^^40^ in addition to signaling and recruiting fibroblasts and (myo)fibroblasts to the site.^41^^,^^42^ These cells then deposit collagen matrix, forming an α-SMA–positive capsule that coats and barricades the foreign body from the host,^19^^,^^36^^,^^43^ which can be similar to synovial-like metaplasia in nature, enabling capsular contracture.^19^^,^^36^^,^^43^^,^^44^ A recent study by Prantl et al^19^ has suggested a proportional relationship between the degree of inflammation and the extent and thickness of subsequent capsule formation, suggesting the paramount importance to reduce the degree of inflammatory response related to the breast reconstruction and TE implantation procedure.

There are multiple factors that may contribute to clinical success in reducing inflammation with capsule formation, with the most critical including patient preparation,^45^^,^^46^ as well as improvements in reconstructive technique,^47^^-^49 breast implant technology,^50^ and ADM application.^51^^,^^52^ While patient selection cannot be controlled, choosing the most appropriate ADM, which will play the most powerful role in improving the long-term reconstructive outcome, is the onus of the surgeon. For example, a specific ADM may be chosen on the basis of its ability to integrate with surrounding tissue, provide sufficient mechanical support to the implant, conform to the shape of the breast, minimize the introduction of foreign material, and mitigate inflammation. However, there is currently no clear guidance for how to choose an appropriate ADM.

In this study, we evaluated the in vitro properties of 5 commercially available HADMs, in comparison with native human skin, in an attempt to predict preclinical inflammatory response.

Methods were chosen on the basis of their ability to differentiate collagen structure, solubility, digestibility, and melting temperature, each of which depends on degree of protein nativity or denaturation. Significant denaturation and changes to native matrix proteins, resulting from damaging processing methods, have been shown to elicit an inflammatory response following implant.^25^^,^^34^ Successful processing of tissues to mitigate this response includes the thorough removal of immuno-antigens from the matrix,^26^^,^^53^ while not inducing damage, which may either be recognized as a foreign body or which may induce neoantigen formation and be targeted by the immune system. Multiple lots were used for testing of each HADM in an effort to reduce any potential effect of donor-to-donor variability.

In vitro, equivalence in morphology, collagen solubility, and resistance to enzymatic digestion were observed here among native skin, hydrated e-HADM, and HADM-FD. In contrast, condensed collagen, higher acid-soluble collagen content, and increased enzymatic digestion rate, indicative of collagen denaturation, were observed for the two γ-irradiated products, g-HADM and g-HADM-FD, and ethanol-stored product, EtOH-HADM.

Previous studies have demonstrated the necessity of unwinding portions of the otherwise enzyme-resistant triple helical (native) collagen prior to peptide bond collagenolysis to expose the specific enzyme cleavage sites.^54^^,^^55^ Since denatured or damaged collagen is typically already unwound to some extent, enzyme digestion occurs more rapidly than for native collagen,^56^ as observed in this study. Previous studies have also demonstrated a link between in vitro susceptibility to digestion by collagenase and host response in nonhuman primates.^29^ Likewise, denaturation tends to increase the acid solubility of collagen, which may explain the more rapid digestion and increased solubility of g-HADM, g-HADM-FD, and EtOH-HADM, which appear to be negatively altered from native human dermis. It is well recognized that γ-irradiation of human collagen matrices leads to an altered collagen morphology and decreased mechanical properties,^57^ as well as altered hydration properties.^58^ Not only are these materials more rapidly digested by collagenases due to process-induced matrix damage but they each were also observed to elicit a moderate inflammatory response with increased induction of MMP-1 activity and capsule formation in this study and previous nonhuman primate studies.^25^ Presence of MMP-1 staining in histological sections of these HADMs seemed to correlate with a decrease in mechanical properties, indicating the effect of partial enzymatic digestion (collagenase) in vivo on strength and elastic modulus during the implant period.

Cell culture studies have also shown the activation of macrophages and inflammatory cytokine production in the presence of damaged ADMs.^23^ HADM collagen denaturation temperature did not vary substantially among the products tested, save for the significantly decreased onset melting temperature demonstrated by g-HADM-FD. Results for this method have been shown previously to be affected differentially by collagen damage as well as chemical treatment related to collagen cross-linking.^34^ There were, however, more apparent differences in enthalpy of melting and peak width among the various HADMs. On the basis of these results, it appears that susceptibility to digestion by collagenase as a marker of collagen nativity and stability may be a good indicator for in vivo inflammation.

In vivo, we evaluated the host response to 3 prehydrated HADMs commonly used in breast reconstruction, with particular emphasis on cell infiltration into the HADM, extent of local and systemic inflammatory response to the implant, and overall effect on HADM integrity as judged by mechanical endurance. A mild inflammatory response, consistent with normal wound healing, was observed for implanted prehydrated e-HADM. Minimal increases in antibody production to the implanted e-HADM systemically, as well as low levels of inflammatory cell infiltration (CD-68) and collagenase production (MMP-1), resulting in lack of rapid e-HADM resorption, and a lack of capsule formation, were observed locally at the TE junction. These results agree with similar findings for HADM-FD previously implanted in the same model^25^ due to the undamaged nature and equivalent processing regimen for these two HADMs. On the basis of the equivalence of e-HADM and HADM-FD in vitro and in vivo, we would predict equivalent responses to be observed for these two products clinically.

Both EtOH-HADM and g-HADM elicited moderate inflammatory responses with differing temporal effects following implantation. This is most likely due to the damaged/denatured state of these HADMs due to processing and/or γ-ray sterilization, which is known to alter collagen structure,^57^ potentially leading to induction of an immune response. For g-HADM, antibody titers peaked at, or just prior to, conclusion of the study and were corroborated by active robust inflammatory cell infiltration into the g-HADM and subsequent capsule formation at the TE junction. For EtOH-HADM, antibody titer peaked earlier and subsided by 10 weeks, suggesting an acute inflammatory response that may have led to rapid resorption/degradation of EtOH-HADM. For both materials, the significant decrease in elastic modulus of both materials following implantation was supported by substantial collagenase (MMP-1) activity and degradation of collagen within the matrix in vivo. In addition, chronic inflammation was indicated by the formation of numerous foreign body giant cells. For EtOH-HADM–implanted animals, the foreign body response to the TE itself was observed in the form of macrophage-concentrated synovial-like hyperplasia and a fibrotic band of capsular tissue. Similar results have been previously published for g-HADM-FD in this same model.^25^

There are hundreds of published articles in the literature pertaining to evaluation of various HADMs. These seek to make comparisons based on small and large animal models and a myriad of different in vitro test methods, some of which appear to illustrate meaningful differences among HADMs. However, the key to test selection is the ability of such a test to predict in vivo, and ultimately, clinical outcomes. The literature is replete with in vitro studies comparing various ADMs, including comparisons of growth factor and DNA content^27^^,^^28^ and out-of-package mechanical properties,^31^ among others. However, these reports have not included corresponding in vivo functional data, either because no correlation was found or because the studies were not conducted. Here, we have shown the in vitro/in vivo correlation between damaged HADMs, as determined by benchtop solubility and rate of enzyme digestion, and resulting nonhuman primate inflammatory response in the presence of a synthetic TE, which is corroborated by previous abdominal wall implantation studies performed without the use of a TE.^29^ Additional studies have also demonstrated the relationship between the presence/absence of matrix biochemical markers as a surrogate for matrix damage^34^^,^^53^ and minimal inflammation in a nonhuman primate implant model. Other studies conducted in nonhuman primates have highlighted the importance of cell-associated immunoantigen removal from ADMs prior to implantation, where matrices positive for alpha-gal^34^ or MHC class I and II antigens^26^ were found to elicit a significant inflammatory response, but those matrices undergoing processing steps to selectively remove cell membrane components elicited only a minimal inflammatory response, consistent with normal wound healing.^26^^,^^53^ Differences observed in inflammatory response to cellular versus noncellular human dermal matrices illustrate the importance of cell-associated immunoantigen removal over specific removal of nonantigenic components, such as DNA.^59^

While characterization of the material properties and host response to specific HADMs is insightful, the ultimate test of HADM functionality is in the clinic. Fortunately, there are abundant data in the literature detailing breast reconstruction clinical outcomes with the use of HADM-FD. In fact, a recent meta-analysis from the period of 2010-2015 identified approximately 275 published clinical articles using HADM-FD and 8 studies with specific mention of e-HADM clinical use.^60^ Studies have shown advances in surgical technique and the use of HADMs have had considerable impact on diminishing breast reconstruction complication rates, with studies reporting between 0% and 2.0% capsular contracture rates with implant HADM-FD coverage.^51^^,^^52^^,^^61^^-^64 This finding is supported by the lack of capsule formation, a precursor for capsular contracture, for both e-HADM and HADM-FD in previous^24^^,^^25^ and current preclinical studies. It is also important to note that the use of HADM-FD did not significantly increase the rate of other common breast reconstruction complications, including low rates of skin flap necrosis,^51^^,^^52^^,^^61^^,^^63^ seroma^61^^-^67 or hematoma formation,^51^^,^^62^^,^^63^^,^^67^ or eventual expander/implant removal.^51^^,^^52^^,^^61^^-^63^,^^65^^,^^67^ A meta-analysis of articles comparing HADM-FD and e-HADM in the same study^68^^-^70 has shown equivalent complication rates.^60^ A report by Khansa et al^71^ reported similar outcomes for e-HADM, and these results are consistent with what we observed in the nonhuman primate model utilized in this study.

Conversely, published breast reconstruction outcomes using g-HADM are limited and have mixed results. Two clinical studies detailing mostly bilateral reconstruction in 10 patients each with g-HADM^72^^,^^73^ report different complication rates, with the Vashi^72^ study having similar seroma and infection rates as HADM-FD and the Bullocks^73^ study reporting significantly higher rates of seroma (22%), infection (11%), and flap necrosis (17%).

A third study with 15 patient enrollment^41^ undergoing unilateral (9 patients) or bilateral reconstruction (6 patients) focused on histological observation at the time of TE-to-implant exchange. While biopsy evaluation appeared to reveal some decrease in both inflammatory cell presence and capsule-like tissue formation with the usage of g-HADM at the TE interface, some degree of α-SMA–positive capsule formation was observed in all operated breasts. Independently conducted head-to-head clinical studies comparing EtOH-HADM with HADM-FD outcomes revealed consistently higher rates of infection, seroma formation, and implant loss for EtOH-HADM reconstructed breasts than for HADM-FD ones,^74^^-^76 which supports the inflammatory-associated results observed in this study.

Here, we have drawn a correlation between benchtop collagen matrix damage and host response in a nonhuman primate preclinical TE model and believe that these results are predictive of clinical outcomes, based on extensive clinical evidence and low inflammation-associated complication rates observed with HADM-FD- and e-HADM–based breast reconstruction, as compared with higher inflammation-associated preclinical and clinical outcomes for EtOH–HADM, which has exhibited evidence of matrix alterations and damage. With limited data availability for g-HADM, it is difficult to make a definitive judgment as to whether this newer HADM positively affects breast reconstruction outcomes, particularly with regard to capsular contracture which was not reported. However, it is believed that with continued usage, g-HADM will provide further evidence of inflammation-associated outcomes, based on the benchtop and primate preclinical observations demonstrated here.

## CONCLUSION

Here, we have shown that demonstrated loss of native collagen integrity in vitro may be predictive of inflammation and capsule formation in a nonhuman primate TE model. The relevance of HADM testing in a model that is immunologically similar to humans, such as the Caribbean vervet primate, is of critical importance, as it will logically be the most predictive of clinical results. It is assumed that, based on the in vivo predictive nature of the benchtop tests used in this study, results from the in vitro tests will also reflect clinical results. However, for those products with little clinical data, this remains to be seen.

## Figures and Tables

**Table 1:** Change in mechanical properties of HADMs following preclinical implantation*

	Maximum stress (MPa)			Elastic modulus (MPa)		
Material	OOP	10 wk’ implant	% Retained	OOP	10 wk’ implant	% Retained
Native HCDM	12.20 (3.51)	N/A	N/A	52.17 (11.98)	N/A	N/A
e-HADM	12.96 (4.65)	7.62 (2.19)^†^	58.83	57.65 (20.24)	44.84 (23.87)	77.78
g-HADM	11.87 (2.02)	7.90 (1.09)^†^	66.58	37.43 (7.52)^‡^	19.58 (1.16)^†^	52.31
EtOH-HADM	19.63 (3.01)†	5.21 (1.57)^†^	26.51	61.15 (9.12)	17.92 (4.02)^†^	29.30

*Maximum stress (MPa) and elastic modulus (MPa) of HADMs both OOP and following 10 weeks’ implantation in the nonhuman primate subcutaneous tissue expander model. Percentage mechanical properties retained were also calculated to show the extent of change for each HADM material. Statistical differences (*P* < .05) were calculated for comparison between OOP and postimplant mechanical properties as well as comparison of OOP properties with native dermis mechanical properties. Native HCDM indicates unprocessed human cellular dermal matrix (skin); e-HADM, electron beam irradiated, hydrated human acellular dermal matrix; g-HADM, γ-irradiated HADM; EtOH-HADM, ethanol-stored HADM; OOP, out-of-package; and N/A, not applicable.

†*P* < .05, comparison of OOP with 10 weeks’ implant.

‡*P* < .05, comparison of OOP with native HCDM.

## References

[1] Eric M, Mihic N, Krivokuca D (2009). Breast reconstruction following mastectomy; patient's satisfaction. Acta Chir Belg.

[2] Tachi M, Yamada A (2005). Choice of flaps for breast reconstruction. Int J Clin Oncol.

[3] Crespo LD, Eberlein TJ, O'Connor N, Hergrueter CA, Pribaz JJ, Eriksson E (1994). Postmastectomy complications in breast reconstruction. Ann Plast Surg.

[4] Noda S, Eberlein TJ, Eriksson E (1994). Breast reconstruction. Cancer.

[5] Moyer KE, Ehrlich HP (2013). Capsular contracture after breast reconstruction: collagen fiber orientation and organization. Plast Reconstr Surg.

[6] Duxbury PJ, Harvey JR (2016). Systematic review of the effectiveness of polyurethane-coated compared with textured silicone implants in breast surgery. J Plast Reconstr Aesthet Surg.

[7] Headon H, Kasem A, Mokbel K (2015). Capsular contracture after breast augmentation: an update for clinical practice. Arch Plast Surg.

[8] Alderman AK, Wilkins EG, Kim HM, Lowery JC (2002). Complications in postmastectomy breast reconstruction: two-year results of the Michigan Breast Reconstruction Outcome Study. Plast Reconstr Surg.

[9] Spear SL (2007). Reoperations or revisions. Plast Reconstr Surg.

[10] Cordeiro PG, McCarthy CM (2006). A single surgeon's 12-year experience with tissue expander/implant breast reconstruction: part I: a prospective analysis of early complications. Plast Reconstr Surg.

[11] Steiert AE, Boyce M, Sorg H (2013). Capsular contracture by silicone breast implants: possible causes, biocompatibility, and prophylactic strategies. Med Devices.

[12] del Pozo JL, Tran NV, Petty PM (2009). Pilot study of association of bacteria on breast implants with capsular contracture. J Clin Microbiol.

[13] Komorowska-Timek E, Oberg KC, Timek TA, Gridley DS, Miles DA (2009). The effect of AlloDerm envelopes on periprosthetic capsule formation with and without radiation. Plast Reconstr Surg.

[14] Wang J, Hori K, Ding J (2011). Toll-like receptors expressed by dermal fibroblasts contribute to hypertrophic scarring. J Cell Physiol.

[15] Barr S, Hill E, Bayat A (2009). Current implant surface technology: an examination of their nanostructure and their influence on fibroblast alignment and biocompatibility. ePlasty.

[16] Barr S, Hill E, Bayat A (2010). Patterning of novel breast implant surfaces by enhancing silicone biocompatibility, using biomimetic topographies. ePlasty.

[17] Jr Adams WP, Rios JL, Smith SJ (2006). Enhancing patient outcomes in aesthetic and reconstructive breast surgery using triple antibiotic breast irrigation: six-year prospective clinical study. Plast Reconstr Surg.

[18] Anderson PR, Freedman G, Nicolaou N (2009). Postmastectomy chest wall radiation to a temporary tissue expander or permanent breast implant—is there a difference in complication rates?. Int J Radiat Oncol Biol Phys.

[19] Prantl L, Schreml S, Fichtner-Feigl S (2007). Clinical and morphological conditions in capsular contracture formed around silicone breast implants. Plast Reconstr Surg.

[20] Israeli R, Feingold RS (2011). Acellular dermal matrix in breast reconstruction in the setting of radiotherapy. Aesthet Surg J.

[21] Sbitany H, Serletti JM (2011). Acellular dermis-assisted prosthetic breast reconstruction: a systematic and critical review of efficacy and associated morbidity. Plast Reconstr Surg.

[22] Kim JY, Davila AA, Persing S (2012). A meta-analysis of human acellular dermis and submuscular tissue expander breast reconstruction. Plast Reconstr Surg.

[23] Orenstein S, Qiao Y, Kaur M, Klueh U, Kreutzer D, Novitsky Y (2010). In vitro activation of human peripheral blood mononuclear cells induced by human biologic meshes. J Surg Res.

[24] Stump A, Holton LH, Connor J, Harper JR, Slezak S, Silverman RP (2009). The use of acellular dermal matrix to prevent capsule formation around implants in a primate model. Plast Reconstr Surg.

[25] Sandor M, Singh D, Silverman RP, Xu H, De Deyne PG (2014). Comparative host response of 2 human acellular dermal matrices in a primate implant model. ePlasty.

[26] Xu H, Wan H, Sandor M (2008). Host response to human acellular dermal matrix transplantation in a primate model of abdominal wall repair. Tissue Eng Part A.

[27] Hathaway JK, Choe JM (2002). Intact genetic material is present in commercially processed cadaver allografts used for pubovaginal slings. J Urol.

[28] Gilbert TW, Freund JM, Badylak SF (2009). Quantification of DNA in biologic scaffold materials. J Surg Res.

[29] Sun WQ, Xu H, Sandor M, Lombardi J (2013). Process-induced extracellular matrix alterations affect the mechanisms of soft tissue repair and regeneration. J Tissue Eng.

[30] Majumder A, Scott JR, Novitsky YW (2016). Evaluation of the antimicrobial efficacy of a novel rifampin/minocycline-coated, noncrosslinked porcine acellular dermal matrix compared with uncoated scaffolds for soft tissue repair. Surg Innov.

[31] Deeken CR, Eliason BJ, Pichert MD, Grant SA, Frisella MM, Matthews BD (2012). Differentiation of biologic scaffold materials through physicomechanical, thermal, and enzymatic degradation techniques. Ann Surg.

[32] Pui CL, Tang ME, Annor AH (2012). Effect of repetitive loading on the mechanical properties of biological scaffold materials. J Am Coll Surg.

[33] Schmitz M, Bertram M, Kneser U, Keller AK, Horch RE (2013). Experimental total wrapping of breast implants with acellular dermal matrix: a preventive tool against capsular contracture in breast surgery?. J Plast Reconstr Aesthet Surg.

[34] Sandor M, Xu H, Connor J (2008). Host response to implanted porcine-derived biologic materials in a primate model of abdominal wall repair. Tissue Eng Part A.

[35] Neidert MR, Lee ES, Oegema TR, Tranquillo RT (2002). Enhanced fibrin remodeling in vitro with TGF-beta1, insulin and plasmin for improved tissue-equivalents. Biomaterials.

[36] Anderson JM, Rodriguez A, Chang DT (2008). Foreign body reaction to biomaterials. Semin Immunol.

[37] Stevens WG, Calobrace MB, Harrington J, Alizadeh K, Zeidler KR, d'Incelli RC (2016). Nine-year core study data for Sientra's FDA-approved round and shaped implants with high-strength cohesive silicone gel. Aesthet Surg J.

[38] Spear SL, Murphy DK (2014). Natrelle round silicone breast implants: Core Study results at 10 years. Plast Reconstr Surg.

[39] Meza Britez ME, Caballero Llano C, Chaux A (2012). Periprosthetic breast capsules and immunophenotypes of inflammatory cells. Eur J Plast Surg.

[40] Siggelkow W, Faridi A, Spiritus K, Klinge U, Rath W, Klosterhalfen B (2003). Histological analysis of silicone breast implant capsules and correlation with capsular contracture. Biomaterials.

[41] Yu D, Hanna KR, LeGallo RD, Drake DB (2016). Comparison of histological characteristics of acellular dermal matrix capsules to surrounding breast capsules in acellular dermal matrix-assisted breast reconstruction. Ann Plast Surg.

[42] Segreto F, Carotti S, Tosi D (2016). Toll-like receptor 4 expression in human breast implant capsules: localization and correlation with estrogen receptors. Plast Reconstr Surg.

[43] Wolfram D, Rainer C, Niederegger H, Piza H, Wick G (2004). Cellular and molecular composition of fibrous capsules formed around silicone breast implants with special focus on local immune reactions. J Autoimmun.

[44] Hwang K, Sim HB, Huan F, Kim DJ (2010). Myofibroblasts and capsular tissue tension in breast capsular contracture. Aesthetic Plast Surg.

[45] Klasson S, Nyman J, Svensson H, Velander P (2016). Smoking increases donor site complications in breast reconstruction with DIEP flap. J Plast Surg Hand Surg.

[46] Lee TJ, Oh TS, Kim EK (2016). Risk factors of mastectomy skin flap necrosis in immediate breast reconstruction using low abdominal flaps. J Plast Surg Hand Surg.

[47] Nahabedian MY (2016). Implant-based breast reconstruction following conservative mastectomy: one-stage vs. two-stage approach. Gland Surg.

[48] Harless C, Jacobson SR (2015). Current strategies with 2-staged prosthetic breast reconstruction. Gland Surg.

[49] Colwell AS (2015). Current strategies with 1-stage prosthetic breast reconstruction. Gland Surg.

[50] O'Shaughnessy K (2015). Evolution and update on current devices for prosthetic breast reconstruction. Gland Surg.

[51] Salzberg CA, Ashikari AY, Koch RM, Chabner-Thompson E (2011). An 8-year experience of direct-to-implant immediate breast reconstruction using human acellular dermal matrix (AlloDerm). Plast Reconstr Surg.

[52] Spear SL, Parikh PM, Reisin E, Menon NG (2008). Acellular dermis-assisted breast reconstruction. Aesthetic Plast Surg.

[53] Connor J, McQuillan D, Sandor M (2009). Retention of structural and biochemical integrity in a biological mesh supports tissue remodeling in a primate abdominal wall model. Regen Med.

[54] Chung L, Dinakarpandian D, Yoshida N (2004). Collagenase unwinds triple-helical collagen prior to peptide bond hydrolysis. EMBO J.

[55] Perumal S, Antipova O, Orgel JP (2008). Collagen fibril architecture, domain organization, and triple-helical conformation govern its proteolysis. Proc Natl Acad Sci U S A.

[56] Gorgieva S, Kokol V

[57] Gouk SS, Lim TM, Teoh SH, Sun WQ (2008). Alterations of human acellular tissue matrix by gamma irradiation: histology, biomechanical property, stability, in vitro cell repopulation, and remodeling. J Biomed Mater Res B Appl Biomater.

[58] Sun WQ, Leung P (2008). Calorimetric study of extracellular tissue matrix degradation and instability after gamma irradiation. Acta Biomater.

[59] Il'in KV (1966). [The antigenic properties of DNA entering into the composition of an artificial DNA-protein complex]. Biull Eksp Biol Med.

[60] Macarios D, Griffin L, Chatterjee A, Lee LJ, Milburn C, Nahabedian MY (2015). A meta-analysis assessing postsurgical outcomes between aseptic and sterile AlloDerm regenerative tissue matrix. Plast Reconstr Surg Glob Open.

[61] Namnoum JD (2009). Expander/implant reconstruction with AlloDerm: recent experience. Plast Reconstr Surg.

[62] Bindingnavele V, Gaon M, Ota KS, Kulber DA, Lee DJ (2007). Use of acellular cadaveric dermis and tissue expansion in postmastectomy breast reconstruction. J Plast Reconstr Aesthet Surg.

[63] Salzberg CA (2006). Nonexpansive immediate breast reconstruction using human acellular tissue matrix graft (AlloDerm). Ann Plast Surg.

[64] Vardanian AJ, Clayton JL, Roostaeian J (2011). Comparison of implant-based immediate breast reconstruction with and without acellular dermal matrix. Plast Reconstr Surg.

[65] Nahabedian MY (2009). AlloDerm performance in the setting of prosthetic breast surgery, infection, and irradiation. Plast Reconstr Surg.

[66] Spear SL. Discussion (2009). Acellular dermis-assisted prosthetic breast reconstruction versus complete submuscular coverage: a head-to-head comparison of outcomes. Plast Reconstr Surg.

[67] Colwell AS, Damjanovic B, Zahedi B, Medford-Davis L, Hertl C, Austen WG (2011). Retrospective review of 331 consecutive immediate single-stage implant reconstructions with acellular dermal matrix: indications, complications, trends, and costs. Plast Reconstr Surg.

[68] Weichman K, Wilson S, Broer PN (2014). LOP17: an outcomes based evolution of 800 implant based breasts reconstructions with acellular dermal matrix. (Presented at the Sixth Annual Meeting of the European Plastic Surgery Research Council, August 21-24, 2014, Hamburg, Germany). Plast Reconstr Surg.

[69] Yuen JC, Yue CJ, Erickson SW (2014). Comparison between freeze-dried and ready-to-use AlloDerm in alloplastic breast reconstruction. Plast Reconstr Surg Glob Open.

[70] Buseman J, Wong L, Kemper P (2013). Comparison of sterile versus nonsterile acellular dermal matrices for breast reconstruction. Ann Plast Surg.

[71] Khansa I, Hendrick RG Jr, Shore A, Meyerson J, Yang M, Boehmler JH IV (2014). Breast reconstruction with tissue expanders: implementation of a standardized best-practices protocol to reduce infection rates. Plast Reconstr Surg.

[72] Vashi C (2014). Clinical outcomes for breast cancer patients undergoing mastectomy and reconstruction with use of DermACELL, a sterile, room temperature acellular dermal matrix. Plast Surg Int.

[73] Bullocks JM (2014). DermACELL: a novel and biocompatible acellular dermal matrix in tissue expander and implant-based breast reconstruction. Eur J Plast Surg.

[74] Brooke S, Mesa J, Uluer M (2012). Complications in tissue expander breast reconstruction: a comparison of AlloDerm, DermaMatrix, and FlexHD acellular inferior pole dermal slings. Ann Plast Surg.

[75] Liu DZ, Mathes DW, Neligan PC, Said HK, Louie O (2014). Comparison of outcomes using AlloDerm versus FlexHD for implant-based breast reconstruction. Ann Plast Surg.

[76] Ranganathan K, Santosa KB, Lyons DA (2015). Use of acellular dermal matrix in postmastectomy breast reconstruction: are all acellular dermal matrices created equal? *Plast Reconstr Surg*.

